# Utility of Flexible Bronchoscopy for Airway Foreign Bodies Removal in Adults

**DOI:** 10.3390/jcm9051409

**Published:** 2020-05-10

**Authors:** Jose N. Sancho-Chust, Virginia Molina, Sandra Vañes, Ana M. Pulido, Lia Maestre, Eusebi Chiner

**Affiliations:** Secció de Pneumologia, Hospital Universitari Sant Joan d’Alacant, Ctra Alacant-València s/n, 03550 Sant Joan d’Alacant, Spain; virginia_molpe@hotmail.com (V.M.); sandravanesbanos@gmail.com (S.V.); pulidawork@gmail.com (A.M.P.); lia_maestre@yahoo.es (L.M.); echinervives@gmail.com (E.C.)

**Keywords:** bronchoscopy, flexible bronchoscopy, airway foreign bodies

## Abstract

Foreign body aspiration is relatively infrequent in adults. Airway foreign bodies (AFBs) can be removed by flexible bronchoscopy (FB) or rigid bronchoscopy (RB). We performed a retrospective analysis of FBs performed in our centre over a 25 year period, focusing on the procedures that revealed an AFB during the examination stage. We recorded demographic data, clinical characteristics and radiological and bronchoscopic findings. During the study period, 12,588 FBs were performed in adults. Airway foreign bodies were identified in 32 of these cases, giving a prevalence of 0.25%. The most frequent clinical presentation was cough, sputum and fever. The most frequent radiological findings were alveolar infiltrates and atelectasis. In 94% of cases, AFBs were removed successfully by FB; RB was necessary in only 6% of cases. There were no FB-related complications. The most common AFB location was the right bronchial tree (69%). We classified AFBs as organic (85%: animal 57%; vegetable 28%), inorganic (6%) and iatrogenic (9%). Bronchial infection occurred in 51% of cases, and *Actinomyces* spp. was the most common causal microorganism. In conclusion, AFBs are a rare entity with nonspecific clinical presentation, most AFBs were organic, and FB is a safe and effective method for AFB removal.

## 1. Introduction

Accidental foreign body aspiration is less common in adults than in children, and there are major differences in clinical presentation between these two populations. Most publications on the topic are case reports; there are very few case series. Determining the exact prevalence of airway foreign bodies (AFBs) is therefore problematic, but they are thought to account for between 0.2% and 0.33% of all bronchoscopies [1,2,3,4,5].

There are documented cases of AFB managed, without much success, by cough manoeuvres, changes in position and ingestion of olive oil or emetics [6].

Endoscopic AFB removal was first described by Gustav Killian, the “father of bronchoscopy”. In 1898, he reported how he had used the first tracheoscope and a pair of forceps to extract a pig bone from a German farmer’s airway [7].

This innovation completely changed the medical management of AFBs, and rigid bronchoscopy (RB) was used successfully for almost a century [8]. After the invention of flexible bronchoscopy (FB) by Shigeto Ikeda, in 1974 Donald Zavala and Mitchell Rhodes described the first successful application of this technique for AFB removal in artificial models and animals (dogs) [9].

The use of FB quickly extended for this indication with case series recording its success [3,10]. In recent years, researchers have explored the use of FB as the method of choice for AFB removal in adults [11]. Studies have demonstrated the efficacy and safety of this technique in inorganic AFB removal which normally involves a greater risk of airway complications [12,13].

The objective of our study was to measure the prevalence of AFBs in adults undergoing bronchoscopy, to describe the clinical and endoscopic characteristics of AFBs and to determine the efficacy and safety of FB for AFB removal.

## 2. Experimental Section

### 2.1. Study Type

This is a retrospective analysis of the database of all bronchoscopies performed in the Pulmonology Department in the period from January 1994 to October 2019 in patients aged over 18 years. The university hospital where the procedures were performed serves a population of some 250,000 inhabitants with slight variations during the study period. The bronchoscopists in our hospital perform bronchoscopies in the morning, and none work out of hours.

We collected data on the FBs that revealed an AFB during the examination stage and then reviewed the patients’ medical records.

### 2.2. Material Used

The material used in the procedures evolved during the rather long study period, owing to technological improvements in the field of respiratory endoscopy.

Five different experienced bronchoscopists performed the examinations, along with the pulmonology residents who intervened as part of their training programme.

The bronchoscopists used different models of flexible bronchoscopes from the brands Pentax© (e.g., EB-1570k and EB15-J10) and Fujifilm© (e.g., EB530H, EB530S and EB530T). Most bronchoscopes had a distal outer diameter of between 49 mm and 54 mm, with a 2.0 mm working channel. The therapeutic bronchoscopes were slightly bigger with a distal outer diameter of around 58 mm and a 2.8 mm working channel.

The instruments used in the FBs to remove the AFBs also varied and included standard bronchial biopsy forceps (both with and without teeth), foreign body forceps (alligator forceps) and foreign body retrieval baskets.

The samples obtained by bronchial suction were sent to the Microbiology Department for the usual staining and cultures. Quantitative cultures showing more than 1.10^5^ UFC were considered to be significant.

After examining the airway and locating the foreign body, the bronchoscopist removed it with the usual technique, securing it with the forceps or basket and removing it by withdrawing the bronchoscope and instrument together. If the AFB had sharp parts, these were directed towards the forceps or basket as far as possible, to minimise damage to the airway walls.

The FBs were performed in the Bronchoscopy Room of the Pulmonology Department with the assistance of the nursing and auxiliary staff. Some patients were put under minimal or conscious sedation with midazolam, usually at a dose of 1 to 5 mg. The local anaesthetic lidocaine was applied topically to the nostrils and with the bronchoscope to the rest of the airway using the “spray-as-you-go” technique [14].

In cases requiring RB, the bronchoscopist used a Wolf© rigid bronchoscope with the standard accessories, including RB forceps which are considerably larger that FB forceps.

The RBs were performed in the operating theatre with the assistance of the usual anaesthesia team, who put the patient under propofol-induced deep sedation.

### 2.3. Data Collection

After identifying the cases with AFB diagnosis, we collected the following data regarding the endoscopic examination: indication of the examination, type of bronchoscope (FB or RB), point of entry, location of AFB, type of instrument used for removal, presence of histological analysis, microbiological results of the samples obtained, need for RB or surgery, and endoscopy-related complications (bleeding, respiratory failure, pneumothorax, cardiac arrest). We identified the foreign body types, classifying them as organic (of animal or vegetable origin), inorganic (minerals, metals, etc.) or iatrogenic (resulting from a medical intervention, e.g., dental prostheses).

Finally, we reviewed the medical records of each patient, collecting data related to age; sex; psychiatric comorbidities (personality disorder, schizophrenia, bipolar disorder); whether aspiration was voluntary; presence of abnormal radiological findings and their location; type and duration of symptoms; and complications due to the AFB, both short-term (pulmonary infections) and long-term (pulmonary sequelae after AFB removal).

### 2.4. Statistical Analysis

We expressed the numerical variables as means with standard deviations and the categorical variables as percentages. For all calculations we used the statistical package IBM SPSS (version 15.0; SPSS; Chicago, IL, USA).

## 3. Results

During the study period of 25 years and 10 months (310 months), 12,588 FBs were performed in adults. Airway foreign bodies were identified in 32 of these cases, giving a prevalence of 0.25%.

Table 1 shows the demographic, clinical and radiological characteristics of the patients with AFB. In most cases, aspiration of a foreign body was an accidental finding. Suspected AFB was the indication for bronchoscopy in only 28% of these cases; the most common indication was pneumonia. There was no case of suspected AFB that could not be verified through FB examination. The most common clinical presentation was the triad of cough, sputum and fever with a mean duration of symptoms of 14 days before presentation. There were abnormal radiological findings in 94% of cases, the most common being alveolar infiltrates and atelectasis. The follow-up examinations showed no late complications of FB.

Table 2 shows the general characteristics of the FBs. In most cases, the bronchoscopist used an instrument designed specifically for foreign bodies (i.e., forceps or basket) to remove the AFB, although standard biopsy forceps were used in 31% of cases. The rate of success of FB for AFB removal was 94%. Flexible bronchoscopy failed in the remaining 6% of cases because the AFB was too large to pass through the glottis. It remained in the bronchial tree and was successfully removed by RB in a later procedure. No cases required surgery, and there were no FB-related complications.

Table 3 shows the location of the AFBs in the airway. The most common location was the right bronchial tree (69% of cases), followed by the left bronchial tree (25%). Only 6% of AFBs were in the trachea or larynx.

**Figure 1:**
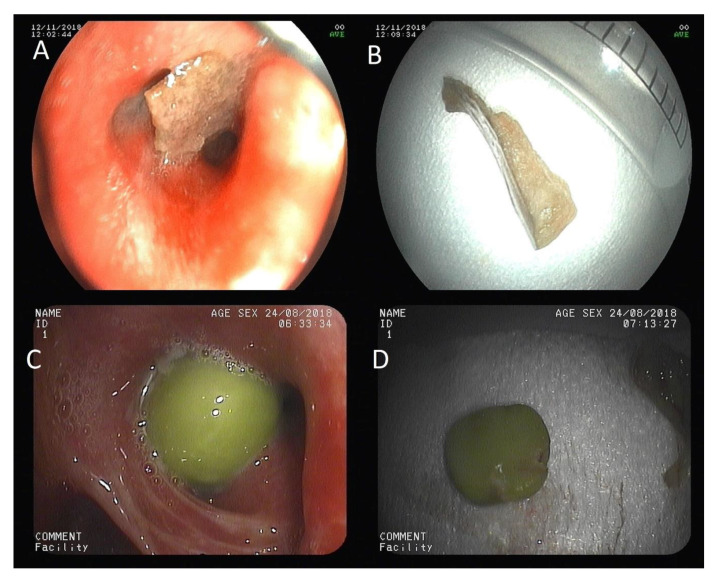
Examples of organic AFBs removed by FB. (**A**) pork rind located in the right lower lobe bronchus; (**B**) pork rind after removal by FB; (**C**) pea located in the left upper lobe bronchus; (**D**) pea after removal. FB: flexible bronchoscopy. AFB: airway foreign body.

Table 4 shows the types of AFBs removed. Organic AFBs accounted for 85% of cases, and of this group, 57% were of animal origin (mainly bones) and 28% of vegetable origin (mainly seeds). Inorganic AFBs accounted for 6% of cases (a ring and a metal cross). The remaining 9% were iatrogenic AFBs, namely, two dental prosthesis and a dental implant. Figure 1 shows some images of AFBs removed by FB.

Pulmonary infection occurred in 51% of cases. Table 5 shows the microorganisms isolated in the FB samples. The most common microorganism was *Actinomyces* spp. which caused 9% of infections, followed by *S. constellatus*, *S. aureus*, *M. morgagni and E. faecium*, each accounting for 6%. We did not observe unusual patterns of resistance: the bacteria were susceptible to the antibiotics most commonly prescribed (beta-lactams, cephalosporins and quinolones). The microbiological results of the bronchoscopy changed the antibiotic indication in only 16% of cases.

## 4. Discussion

The prevalence of AFBs in this study was 0.25% of the bronchoscopies performed in adults. This figure is very similar to those available in the literature. A relatively recent bibliographical review reported a pooled prevalence of 0.24% (95% CI 0.18–0.31), although the proportions in the different studies ranged from 0.16% to 0.33% [1]. These data show that although foreign body aspiration is uncommon in the adult population, it is a potential finding of an endoscopic airway examination. In a medium-sized unit, which performs around 500 bronchoscopies every year, there will be 1 or 2 yearly cases of AFB. Bronchoscopists therefore need to know how to manage this situation.

Different methods are available for removing AFBs. The preferred technique is normally RB, owing to factors such as better airway control, better protection of the mucous membranes, better management in central locations and larger instruments [15]. In recent years, however, more and more case series and systematic reviews have supported FB as the technique of choice, as it is simpler and generally more accessible, affords a much more complete examination of the airway and can be used even in patients with cervical injuries or who are intubated [1,2,3,4,11,16,17,18,19]. There are some very complex cases that may require RB owing to factors such as central location, large size or sharp parts. But in the absence of such factors, FB could be the method of choice for AFB extraction in adults.

The success rate of FB in our study was 94% which is relatively high compared to the rates presented in the literature. The reported efficacy of FB in AFB removal varies from 61% to 100%, with a pooled rate of 89.6% (95% CI 86.1–93.2) [1]. The relatively high success rate in our study is all the more relevant when we consider the size of our bronchoscopy unit. It may seem logical that only bronchoscopists in large units can obtain good results in such an infrequent procedure, as they are bound to treat more cases each year; yet in our study, only 6% of cases required subsequent RB to remove the foreign body. This proves that even small- or medium-sized units can achieve good results with FB.

Only 28% of our cases had an indication of suspected AFB. This is almost certainly because our hospital has no bronchoscopists working out of hours, meaning life-threatening cases, which cannot wait to be attended in the usual morning timeslot, are sent urgently to a nearby tertiary hospital for emergency bronchoscopy. In all likelihood, had we analysed these emergency bronchoscopies, the percentage of cases with suspected AFB would be slightly higher.

The safety data were excellent in this study, as no patients suffered FB-related complications. The rate of complications in previous studies is approximately 1%, with bleeding being the most common complication. As well as being effective, then, FB is safe for AFB removal even in medium-sized units, a fact that further justifies the elective use of this technique [1,17].

In our study, most AFBs were removed with instruments specifically designed for foreign bodies: mainly gripping forceps but also baskets. However, up to 31% of cases were successfully resolved with conventional biopsy forceps which shows that the standard equipment of a respiratory endoscopy unit often suffices for this procedure. The devices for AFB removal described in the literature include standard biopsy forceps, forceps specifically designed for foreign bodies (alligator forceps), magnetic probes (for metal objects), several types of metal hooks, baskets (made of metal or synthetic materials), balloon catheters and even cryoprobes [16,17,18].

With FB there is always a risk that the AFB will migrate during removal. Factors such as size, irregularity, hardness and consistency of the foreign body can influence this risk. To prevent migration, bronchoscopists must try to fully surround the AFB if using a basket or apply firm pressure to effectively grip the AFB if using forceps. The main obstacle to take into account when extracting foreign bodies by FB is the glottis, which is the narrowest part of the airway. When the bronchoscope is inserted nasally, the nasal fossae are another critical point. Of course, sharp objects are more likely to damage the airway, meaning the consequences of migration are more serious. Perhaps RB should be the method of choice in these cases [16,17,18].

In our series there were very few cases of sharp AFBs and AFB migration. This may be because, as explained above, our hospital has no bronchoscopists or pulmonologists working out of hours, meaning life-threatening cases are sent urgently to a nearby tertiary hospital. The fact that we could not include emergency bronchoscopies referred to another hospital may constitute selection bias.

Regarding the access route used in FB, bronchoscopists opted for the nasal route in a rather large proportion of cases (39%) for several different reasons. Firstly, FB was indicated for suspected AFBs in only 28% of cases. For bronchoscopic airway examination, we normally chose the nasal entry route because it is associated with various advantages such as lesser lateral mobility of the bronchoscope and better patient tolerance. In most cases, AFBs were found incidentally during the examination. Airway foreign bodies that appeared to be regularly shaped and smaller in diameter than the far end of the bronchoscope were removed through the same nasal route. For AFBs judged to be irregularly shaped or larger than the bronchoscope, in the same procedure the examiner switched to the oral route for removal (58% of cases), as the AFB may not have passed easily through the nasal fossa. The fact that we encountered no failures due to the difficulty removing AFBs through the nasal fossa would appear to support this approach.

With regard to patients’ clinical characteristics, the most common presentation in our study was the triad of cough, sputum and fever which probably explains why the most common indication for bronchoscopy was pneumonia and why the duration of symptoms before presentation was relatively long. There is a danger, therefore, that symptoms can go unnoticed and be wrongly diagnosed as pneumonia, especially in older patients [20].

Most of our patients had radiological findings of alveolar infiltrate or atelectasis. While both findings are direct consequences of AFBs, there was a radiological suspicion of AFBs in only 18% of cases. In previous studies, this figure ranged from 25% to 47% of cases [1,4]. The nature and size of the foreign objects identified in each series could explain these differences: large and calcific density foreign bodies are easier to detect in a radiological examination than small or vegetable foreign bodies.

In this study, as in previous studies, most foreign objects were located in the right bronchial tree. Anatomical factors explain this universal tendency: the right main bronchus is wider and more vertical than the left main bronchus, and the carina lies left of the trachea midline [21].

Most AFBs found in our study were organic (more animal than vegetable); inorganic and iatrogenic foreign bodies were relatively infrequent. Although the numbers vary in the literature, inorganic and iatrogenic AFB are usually more common than organic AFB [1,3,4,5,6,10]. Two factors could explain our unusual results: firstly, our unit is not a specialised interventional pulmonology unit; and secondly, we do not have an on-call bronchoscopist. Specialised unit case series normally include more cases of inorganic and iatrogenic AFBs because these cases are referred to tertiary referral hospitals. Social and dietary factors associated with the place where the hospital is located may also influence the frequency of different AFB types. Finally, we must keep in mind the inherent variability of case series which usually include dozens of cases [1].

In our study, bronchial infection was reported in 51% of cases. The few studies that include this parameter report similar figures [4]. We considered the pulmonary infection to be relevant in all cases, because significant quantities of microorganisms were isolated with a quantitative culture technique and these findings were consistent with imaging test results and clinical features. Although bronchial infection is rarely considered in the literature, we consider it to be a very significant factor. Our findings in this regard show that foreign-body bronchial obstruction frequently causes infection, probably because it can inhibit natural defence mechanisms of the mucosa and airway such as coughing and ciliary beating. The types of microorganisms identified also suggest that they were probably related to the aspirated content. All patients with a pneumonia diagnosis had taken antibiotics before the bronchoscopy, which could have influenced the rate of positive cultures.

The microbiological results changed the antibiotic indication in only 16% of cases, since in the rest, antibiograms showed that the bacteria were susceptible to the empirically prescribed antibiotic. All this suggests that beta-lactam antibiotics constitute the best empirical treatment for these cases. Including bronchial infection highlights the utility of FB for microbiological diagnoses and can provide useful data on antibiotic resistance in each case, potentially improving patient prognosis.

## 5. Conclusions

The prevalence of AFBs was 0.25% of the bronchoscopies performed in adults.

We found FB to be a safe and effective technique for removing foreign bodies from the airways, with a success rate of 94% and no associated complications.

The most common clinical presentation was the triad of cough, sputum and fever. The most frequent radiological findings were alveolar infiltrates and atelectasis.

Most AFBs were organic; inorganic and iatrogenic AFBs were relatively infrequent.

Bronchial infection occurred in around half of cases.

## Figures and Tables

**Table 1:** Clinical and radiological characteristics of patients with airway foreign bodies (AFBs).

Parameter	Value
Age	63 ± 12 (32–81)
Men	53%
Psychiatric comorbidities	25%
Voluntary foreign body aspiration	6%
Bronchoscopy indication:	
- Pneumonia	35%
- Suspected AFB	28%
- Atelectasis	28%
- Suspected lung cancer	3%
- Haemoptysis	3%
- COPD	3%
Clinical presentation:	
- Cough, sputum and fever	34%
- Dyspnoea	25%
- Cough	22%
- No symptoms	16%
- Haemoptysis	3%
Duration of symptoms before presentation	14 ± 17 (0–60)
Radiological findings:	
- Alveolar infiltrate	38%
- Atelectasis	38%
- Calcific density	18%
- No findings	6%
Sequelae after removal	0%

Data are presented as number of patients (%) or mean ± SD (range). COPD: Chronic Obstructive Pulmonry Disease.

**Table 2:** Characteristics of flexible bronchoscopy (FB) in patients with an AFB.

Parameter	Occurrence
Point of entry:	
- Mouth	58%
- Nose	39%
- Tracheostomy	3%
Instrument used:	
- Foreign body forceps	53%
- Standard biopsy forceps	31%
- Foreign body basket	16%
Histological confirmation	13%
Result of FB:	
- Removed	94%
- Failure and need for RB	6%
- Failure and referral to Thoracic Surgery	0%
Complications of the procedure	0%

Data are presented as number of patients (%).

**Table 3:** Location of the foreign bodies in the airway.

Location	Occurrence
Larynx	3%
Trachea	3%
Right main bronchus	13%
Right upper lobe bronchus	3%
Bronchus intermedius	19%
Middle lobe bronchus	6%
Right lower lobe bronchus	28%
Left main bronchus	3%
Left upper lobe bronchus	9%
Left lower lobe bronchus	11%

Data are presented as number of patients (%).

**Table 4:** Classification and specifications of AFB types.

AFB Type	Occurrence
ANIMAL	
Animal bone	48%
Pork lard	3%
Snail	3%
Nail	3%
VEGETABLE	
Seed	
- Pea	10%
- Walnut	6%
- Corn	6%
- Lemon	3%
Carrot	3%
INORGANIC	
Ring	3%
Metal cross	3%
IATROGENIC	
Dental prosthesis	6%
Dental implant	3%

Data are presented as number of patients (%).

**Table 5:** Microbiological isolation in FB samples in patients with AFB.

Microorganism	Occurrence
No microorganism isolated	49%
*Actinomyces* spp.	9%
*S. constellatus*	6%
*S. aureus*	6%
*M. morgagni*	6%
*E. faecium*	6%
*Serratia* spp.	3%
*Acinetobacter baumannii*	3%
*M. pneumoniae*	3%
*Prevotella buccae*	3%
*M. morgagni*	3%
*Candida* spp.	3%

Data are presented as number of patients (%).
